# Supplementary material for: The Anti-Candida Activity of Tephrosia apollinea Is More Superiorly Attributed to a Novel Steroidal Compound with Selective Targeting
Source: Plants (Basel). 2022 Aug 15;11(16):2120. doi: 10.3390/plants11162120 (PMC9415581; doi:10.3390/plants11162120)

Supplementary Data

Spectra of compound 1

S1. ^{1}H NMR spectrum of compound 1

S2. ^{13}C NMR spectrum of compound 1

S3. HSQC spectrum of compound 1

S4. HMBC spectrum of compound 1

S5. COSY spectrum of compound 1

S1. ^{1}H NMR spectrum of compound 1

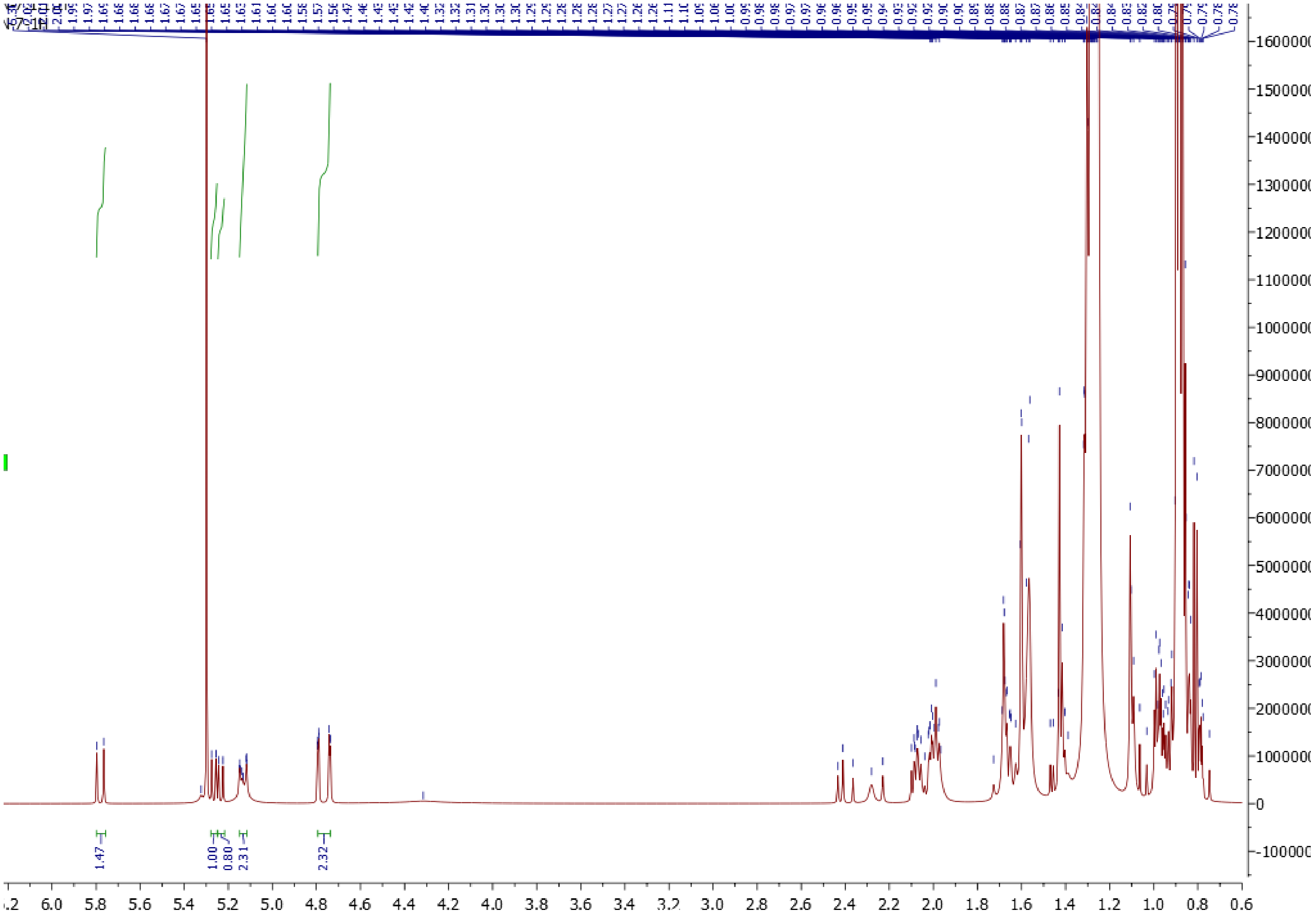

S2. ^{13}C NMR spectrum of compound 1

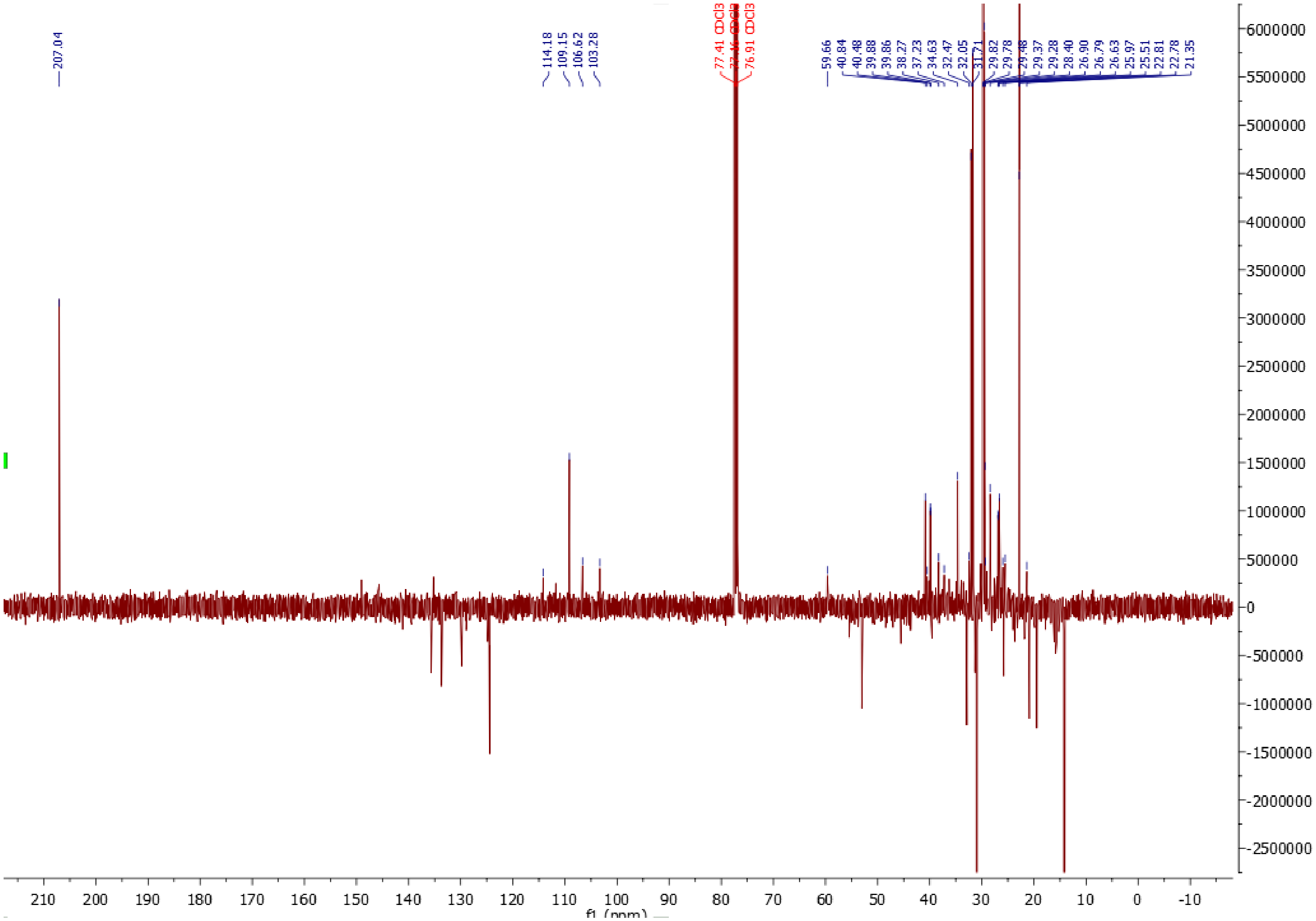

S3. HSQC spectrum of compound 1

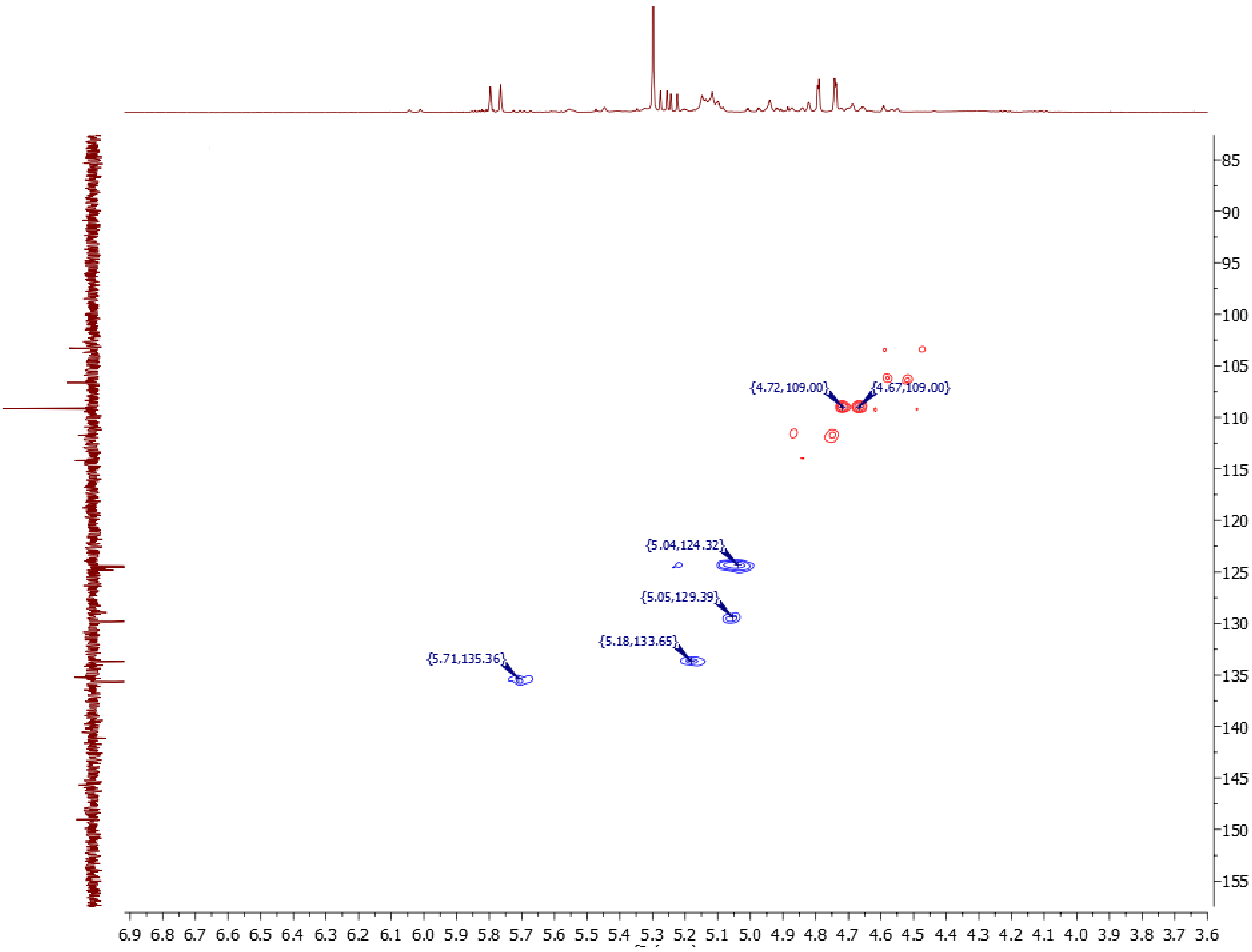

S4. HMBC spectrum of compound 1

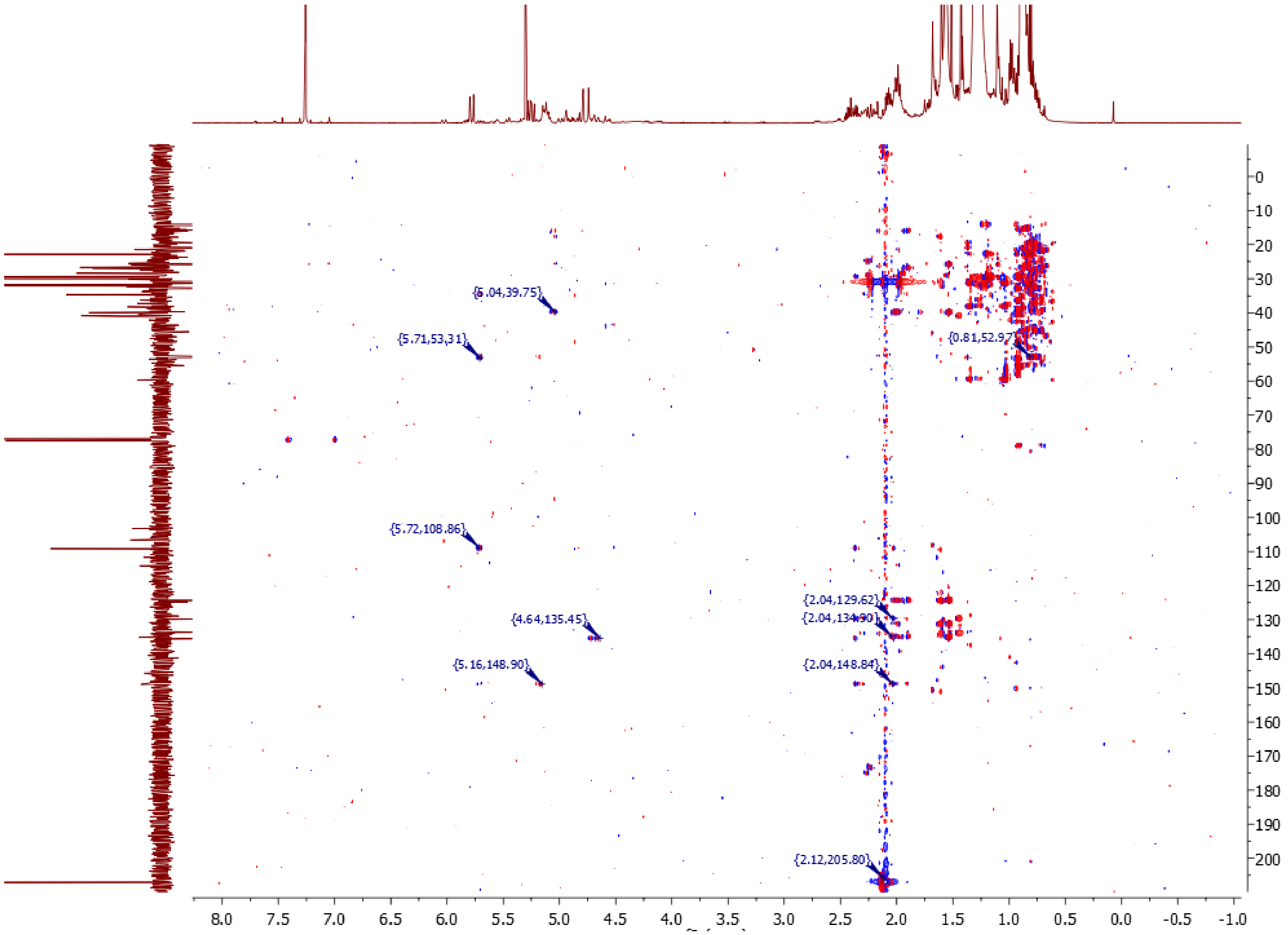

S5. COSY spectrum of compound 1

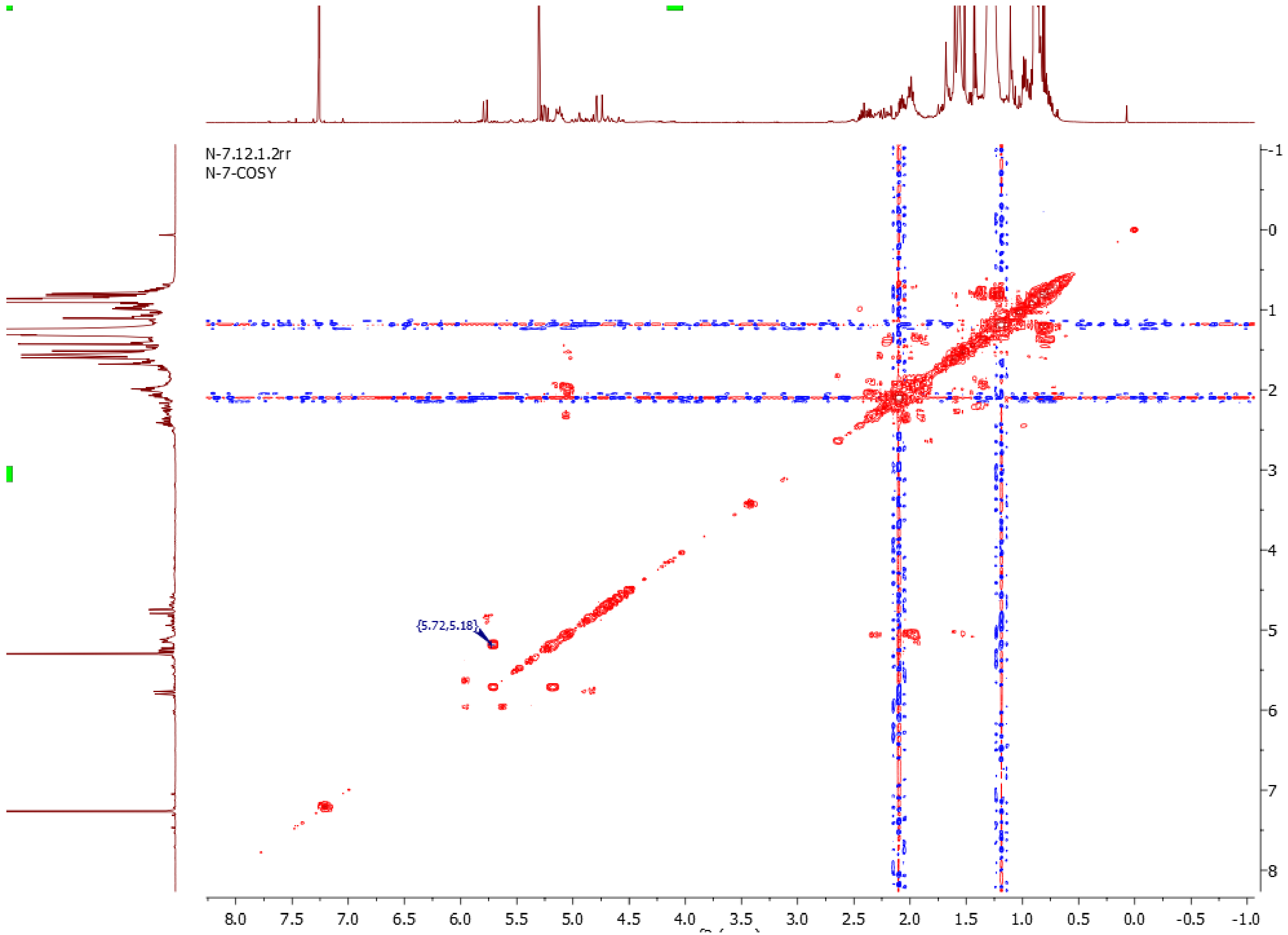

Compound 2: ^{1}H NMR (500 MHz, CD_3OD): δ=7.99 (1H, *d*, *J* = 8.8 Hz, H-5), 7.54 (1H, *d*, *J* = 8.8 Hz, H-6), 7.47-7.45 (2H, *m*, H-2’&6’), 7.42-7.40 (3H, *m*, H-3’&5’&4’), 6.55 (1H, *d*, *J* = 10.07 Hz, H-2’’), 5.92 (1H, *s*, H-3), 5.59 (1H, *d*, *J* = 10.07 Hz, H-1’’), 3.83 (3H, s, OCH_3), 1.48 (3H, *s*, H-4’’), 1.31 (3H, *s*, H-5’’).

Spectra of compound 2

S6. ^{1}H NMR spectrum of compound 2

S7. COSY spectrum of compound 2

S6. HNMR spectrum of compound 2

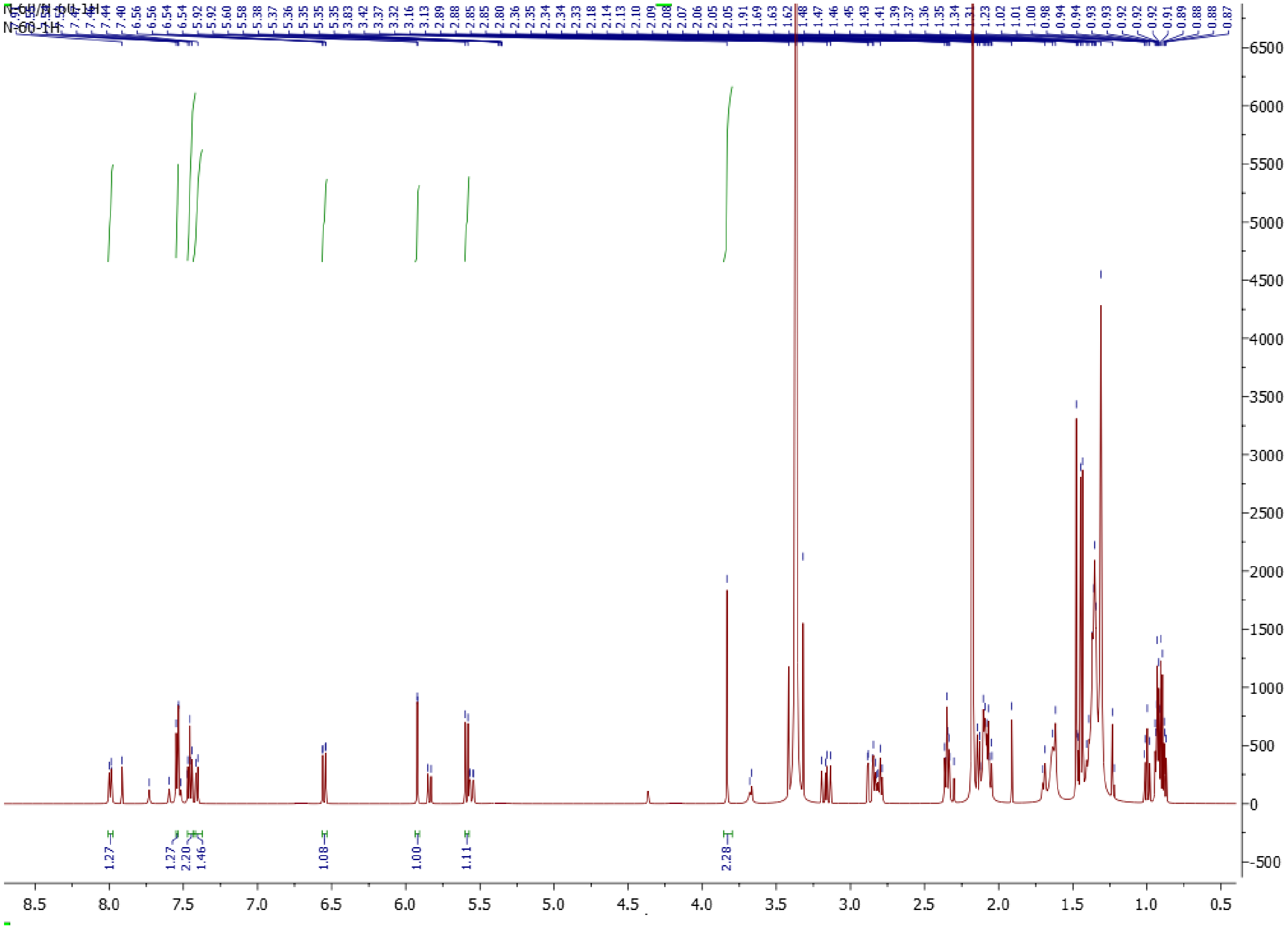

S7. COSY spectrum of compound 2

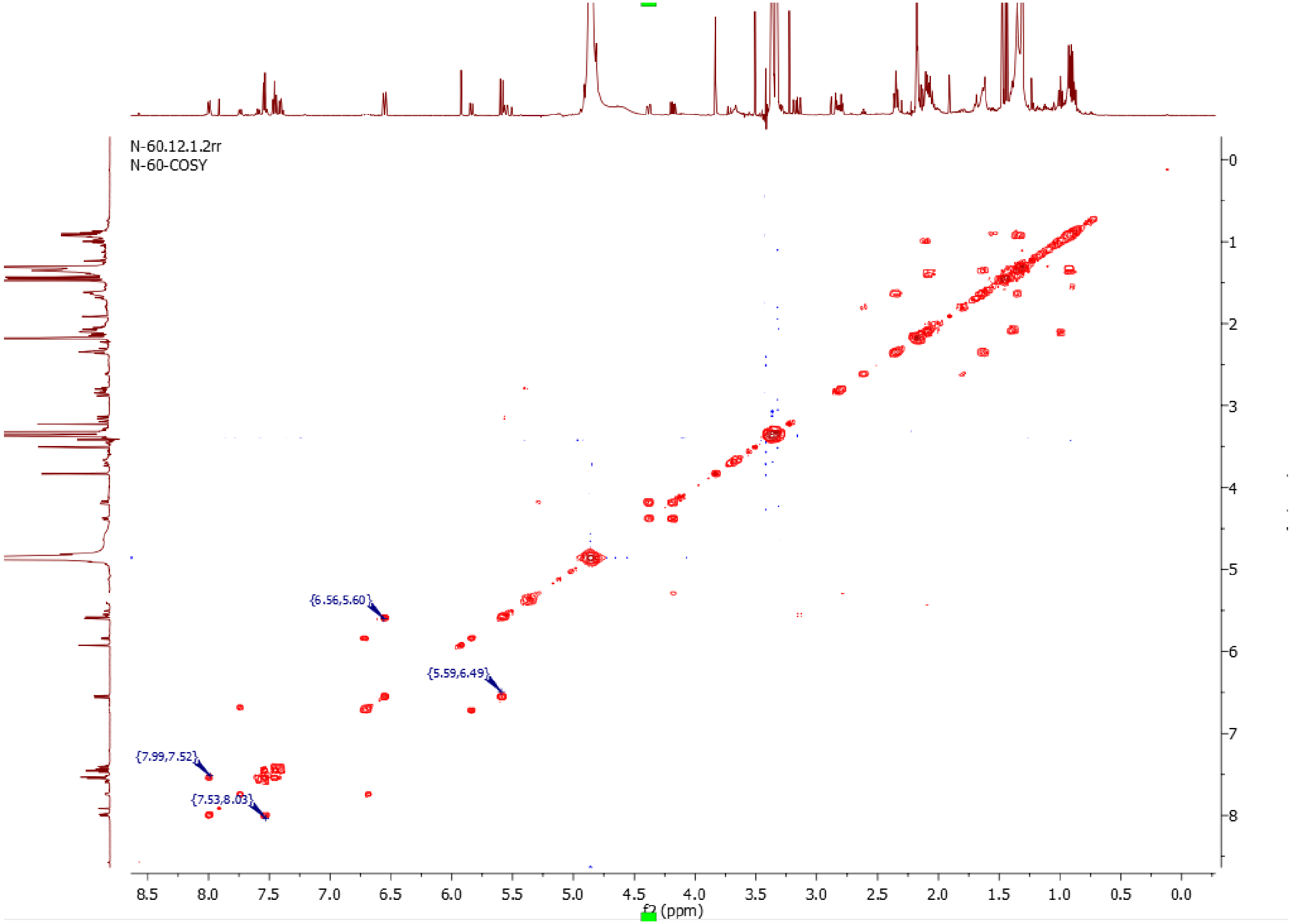

Compounds 3 and 4: ^{1}H NMR spectral analyses (500 MHz, $CDCl_3$) δ= 5.28 (1H, *t*, H-6), 3.45 (1H, *m*, H-3), 1.92 (2H, *m*, H-7), 1.77, 1.02 (2H, *m*, *m*, H-1), 1.77, 1.19 (2H, *m*, *m*, H-16), 1.60 (1H, *m*, H-25), 1.47, 0.98 (2H, *s*, *m*, H-15), 1.41 (2H, *m*, H-2), 1.28 (1H, *m*, H-20), 1.23, 0.94 (2H, *m*, *s*, H-22), 1.09 (2H, *m*, H-23), 0.93 (3H, *s*, H-19), 0.85 (1H, *d*, *J* = 6.5 Hz, H-9), 0.85 (3H, *d*, *J* = 6.5 Hz, H-21), 0.85 (1H, *d*, *J* = 6.5 Hz, H-24), 0.78 (3H, *d*, *J* = 7.5 Hz H-27), 0.76 (3H, *t*, H-29), 0.74 (3H, *d*, *J* = 7.5 Hz, H-26), 0.61(3H, *s*, H-18). ^{13}C NMR spectral analyses (100 MHz, $CDCl_3$) showed the following signals (δ ppm): 140.9 (C-5), 121.8 (C-6), 72.0 (C-3), 56.8 (C-14), 56.2 (C-17), 50.3 (C-9), 46.01 (C-24), 42.4 (C-4), 42.4 (C-13), 39.9 (C-12), 37.4 (C-1), 36.7 (C-10), 36.3 (C-20), 34.1 (C-22), 32.1 (C-7), 32.1 (C-8), 31.8 (C-2), 29.3 (C-25), 28.4 (C-16), 26.3 (C-23), 24.5 (C-15), 23.2 (C-28), 21.2 (C-11), 19.9 (C-27), 19.6 (C-26), 19.2 (C-19), 19.0 (C-21), 12.1 (C-29), 12.0 (C-18).

Spectra of compound 3 & 4

S8. ^{1}H NMR spectrum of compounds **3** & **4**

S9. ^{13}CNMR spectrum of compounds **3** & **4**

S8. ^{1}H NMR spectrum of compounds **3** & **4**

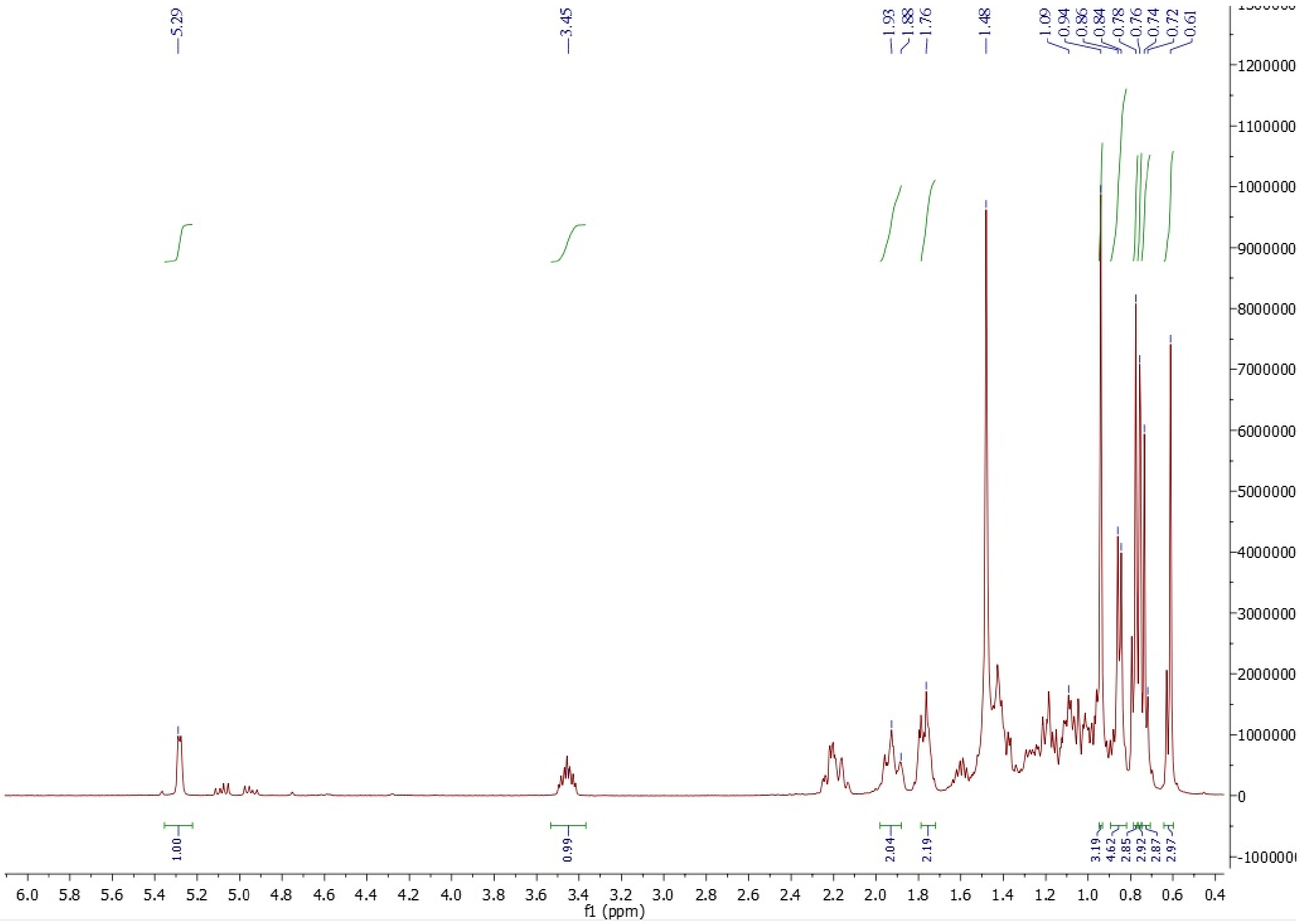

S9. ^{13}CNMR spectrum of compounds **3** & **4**

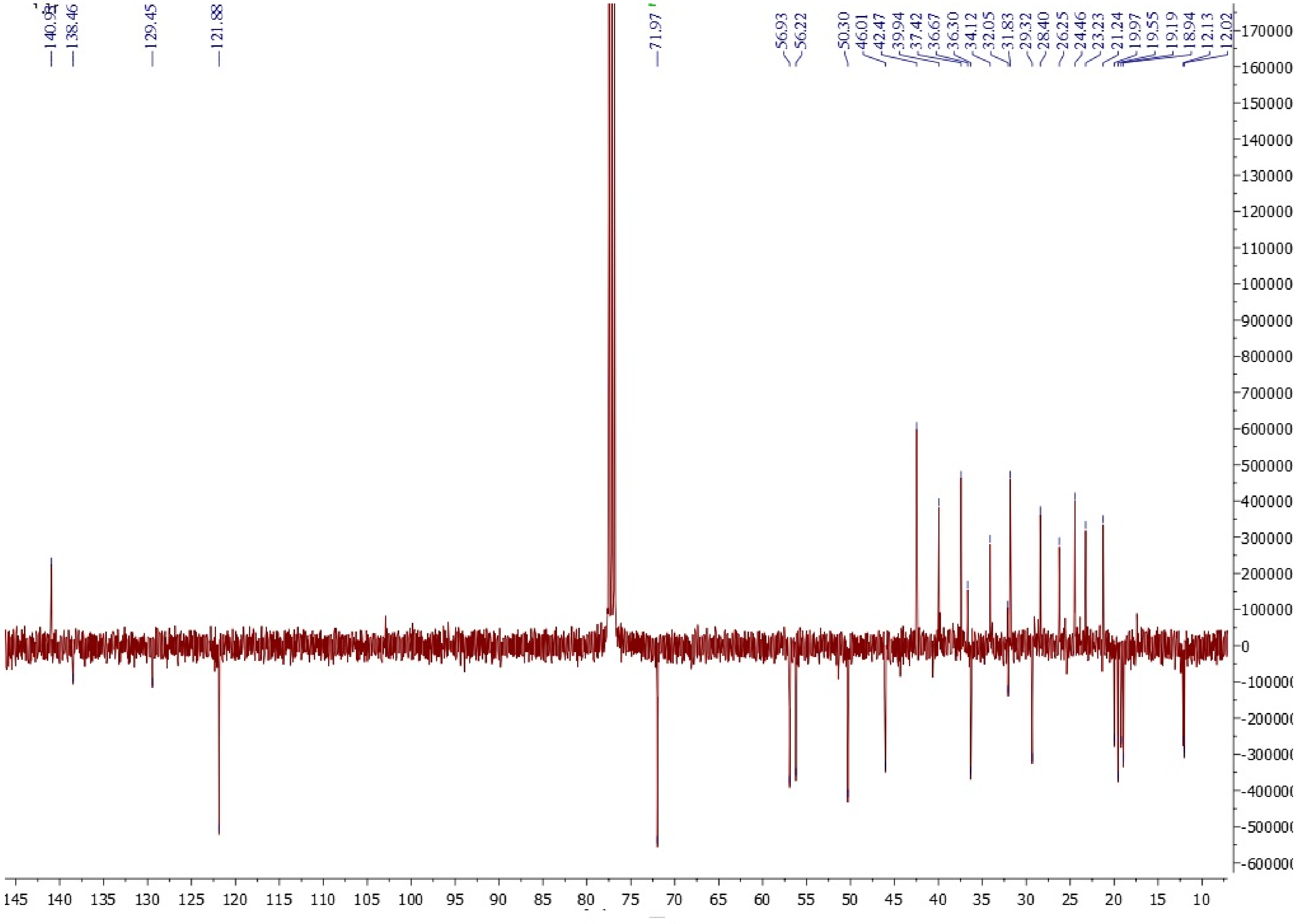

Supplement: Supplementary file 1 [file plants-11-02120-s001.zip › plants-1829389-supplementary.pdf]
